# The burden of childhood hand-foot-mouth disease morbidity attributable to relative humidity: a multicity study in the Sichuan Basin, China

**DOI:** 10.1038/s41598-020-76421-7

**Published:** 2020-11-10

**Authors:** Caiying Luo, Yue Ma, Yaqiong Liu, Qiang Lv, Fei Yin

**Affiliations:** 1 West China School of Public Health and West China Fourth Hospital, Sichuan University, Chengdu, Sichuan China; 2 Sichuan Center for Disease Control and Prevention, Chengdu, Sichuan China

**Keywords:** Infectious diseases, Public health, Epidemiology, Environmental health

## Abstract

Hand, foot and mouth disease (HFMD) is a growing threat to children's health, causing a serious public health burden in China. The relationships between associated meteorological factors and HFMD have been widely studied. However, the HFMD burden due to relative humidity from the perspective of attributable risk has been neglected. This study investigated the humidity-HFMD relationship in three comprehensive perspectives, humidity-HFMD relationship curves, effect modification and attributable risks in the Sichuan Basin between 2011 and 2017. We used multistage analyses composed of distributed lag nonlinear models (DLNMs), a multivariate meta-regression model and the calculations of attributable risk to quantify the humidity-HFMD association. We observed a J-shaped pattern for the pooled cumulative humidity-HFMD relationship, which presented significant heterogeneity relating to the geographical region and number of primary school students. Overall, 27.77% (95% CI 25.24–30.02%) of HFMD infections were attributed to humidity. High relative humidity resulted in the greatest burden of HFMD infections. The proportion of high humidity-related HFMD in the southern basin was higher than that in the northern basin. The findings provide evidence from multiple perspectives for public health policy formulation and health resource allocation to develop priorities and targeted policies to ease the HFMD burden associated with humidity.

## Introduction

Hand, foot, and mouth disease (HFMD) is a common acute infection caused by a series of enteroviruses and coxsackie viruses^1^. It mostly occurs in children under 5 years old. The disease is mainly transmitted via the fecal–oral route, followed by the respiratory route^2^. The clinical symptoms in most patients are mild, self-limited and generally tend to disappear or decrease in 7–10 days. Some patients, however, may rapidly develop severe complications, such as central nervous system damage, cardiopulmonary failure, and even death^3^. In the past few decades, several HFMD epidemics and outbreaks have been reported in the Asian-Pacific region^4–6^. In China, HFMD, a primary infectious disease, has the highest annual incidence, ranging from 37.01/100,000 to 205.06/100,000^7^, compared with all the other notifiable infectious diseases (such as influenza, with an incidence of 14.37/100,000 to 55.09/100,000). Moreover, since 2008, the number of reported HFMD cases has been increasing dramatically, and the cumulative number has reached over 22 million, surpassing the numbers of cases in most Asian-Pacific countries^8^. Based on the severity of the HFMD situation and its growing threat to children, HFMD has recently become an important public health issue that has attracted much attention.

Many epidemiological studies have revealed that meteorological factors tend to have complex nonlinear and delayed effects on HFMD^9–15^. The majority of studies focused on exploring the impacts of temperature and relative humidity on HFMD infections. Numerous studies analyzed and found a consistent relationship between temperature and HFMD. However, for the effects of relative humidity, the existing literature has suggested inconsistent results. For example, early studies generally showed a simple linear relationship. A^16^ study in Beijing showed that relative humidity was negatively associated with HFMD infections. In contrast, other studies from Thailand^17^, the Mekong Delta region in Vietnam^18^, Taiwan^19^, Shandong^20^ and Guangdong^21^ showed positive associations. Recently, epidemiologists have shown increasing interest in explicating potential complicated nonlinear impacts of relative humidity. A study in Hong Kong^22^ revealed a J-shaped relationship, while another study in Guangdong^23^ found an increasing risk of HFMD with relative humidity, with 86.9% relative humidity at the peak of the exposure–response curve. The heterogeneity of results might be associated with the differences in geographic locations, socioeconomic statuses and health services.

Most previous studies constructed single-city time series regressions with different modeling approaches and parameter determinations, which limit the comparability of city-specific findings. Diverse city-level factors may also cause inconsistent results across different cities, limiting generalizability. Therefore, a multicity distributed lag nonlinear modeling approach should be developed in which relationships in each city are assessed, and then the city-specific effects are pooled to explore the source of the heterogeneous results^24^. Moreover, the impacts of relative humidity on HFMD were primarily quantified by measuring risk ratios (e.g., RR or OR) in published studies^25–27^, and the extra burden of HFMD attributed to exposure was not adequately assessed. Risk ratios and similar effect estimates are more difficult to understand from a public health perspective, while attributable numbers or fractions are easier to comprehend. Thus, calculating attributable risk indexes, such as the attributable number (AN) or attributable fraction (AF), is appropriate to reflect the overall disease burden associated with exposure factors and provide new evidence for effective interventions, health resource allocation, and predictions of the effects of climate change. Very few studies have explored the morbidity burden of HFMD infection attributable to relative humidity. To the best of our knowledge, no studies have focused on revealing the attributable risk in consideration of the possible complex nonlinear impacts and delayed impacts in the humidity-HFMD relationship. Therefore, the present study quantified the attributable risks of HFMD due to relative humidity based on the complex non-linear and lagged exposure–response relationship in the Sichuan Basin, an area with diverse climatic and socioeconomic characteristics among cities, which provides a more comprehensive and rational insight into the association.

The Sichuan Basin was chosen as our research area based on the following two reasons. First, the Sichuan region has been experiencing a severe HFMD epidemic since 2009, and the annual incidence continues to increase. In 2018, more than 13 thousand cases were reported, which was 6 times higher than that in 2009. Second, the Sichuan Basin has a complex terrain, and the numerous rivers lead to high and variable air humidity. Moreover, 17 cities in the Sichuan Basin have variable degrees of economic development, demographic characteristics, and health services. These factors may modify the effect of relative humidity on HFMD.

We thus aimed to analyze the time series association of relative humidity with HFMD in 17 Sichuan Basin cities between 2011 and 2017, explore the modification effects of city-level variables, and quantify the infection burden of HFMD attributed to relative humidity to narrow the knowledge gap left by previous studies.

## Results

### Data description

A total of 427,788 HFMD cases were reported in the 17 cities in the Sichuan Basin between 2011 and 2017. On average, the daily number of reported HFMD cases was 10 (range 0–33). According to city-specific total numbers of reported HFMD cases, Chengdu, Meishan, Mianyang, and Deyang were the top 4 cities with the highest numbers of cases (see Online Appendix Figure S1). The mean daily relative humidity was 76.6% (range 15–100%), with Luzhou having the highest (83.74%) and Guangyuan having the lowest (68.32%). The daily mean temperature, wind velocity, sunshine duration, and atmospheric pressure were 17.7 °C (range − 2.2 to 35.4 °C), 1.3 m/s (range 0–6.4 m/s), 3.16 h (range 0–13.3 h) and 965.56 hPa (range 748.25–1003.1 hPa), respectively (Table 1). More information on socioeconomic statuses in the 17 cities is shown in Online Appendix Table S1.

**Table 1:** Summary statistics of the city-specific meteorological characteristics and disease counts of HFMD in the Sichuan Basin.

City	Cases	Relative humidity (℃, mean ± sd)	Temperature	Atmospheric pressure (hPa, mean ± sd)	Wind velocity (m/s, mean ± sd)	Sunshine duration (h, mean ± sd)
Chengdu	184,673	79.34 ± 8.64	16.44 ± 7.4	951.03 ± 7.43	1.22 ± 0.48	2.74 ± 3.33
Zigong	7889	81 ± 10.27	17.87 ± 7.52	973 ± 8.18	1.3 ± 0.54	3.01 ± 3.83
Luzhou	8424	83.74 ± 11.52	18.09 ± 7.38	970.86 ± 8.14	1.7 ± 0.52	3.27 ± 4.08
Deyang	22,573	70.7 ± 12.7	17.4 ± 7.6	954.14 ± 7.52	1.71 ± 0.66	3.35 ± 3.7
Mianyang	24,614	70.7 ± 12.7	17.4 ± 7.6	954.14 ± 7.52	1.71 ± 0.66	3.35 ± 3.7
Guangyuan	10,438	68.32 ± 14.35	16.61 ± 7.74	953.91 ± 7.63	1.5 ± 0.77	3.59 ± 3.78
Suining	16,104	77.14 ± 12.62	17.65 ± 7.72	972.8 ± 8.15	1.24 ± 0.52	3.04 ± 3.77
Neijiang	14,197	81 ± 10.27	17.87 ± 7.52	973 ± 8.18	1.3 ± 0.54	3.01 ± 3.83
Leshan	15,979	76.67 ± 10.98	18.22 ± 7.22	964.89 ± 7.91	1.1 ± 0.42	2.85 ± 3.66
Nanchong	19,818	76.75 ± 13.39	18.1 ± 7.88	976.59 ± 8.47	1.44 ± 0.71	3.52 ± 4.04
Meishan	30,687	76.67 ± 10.98	18.22 ± 7.22	964.89 ± 7.91	1.1 ± 0.42	2.85 ± 3.66
Yibin	8996	75.41 ± 11.28	18.79 ± 7.29	972.77 ± 16.44	0.95 ± 0.43	2.79 ± 3.72
Guangan	9527	76.75 ± 13.39	18.1 ± 7.88	976.59 ± 8.47	1.44 ± 0.71	3.52 ± 4.04
Dazhou	16,321	73.85 ± 11.42	18.04 ± 7.95	973.92 ± 8.13	0.94 ± 0.48	3.21 ± 3.97
Yaan	10,226	78.89 ± 10.63	16.81 ± 7.13	942.9 ± 7.12	0.95 ± 0.55	2.42 ± 3.23
Bazhong	13,487	74.2 ± 10.72	17.34 ± 7.89	966.08 ± 7.85	1.13 ± 0.56	4.19 ± 4.3
Ziyang	13,835	81 ± 10.27	17.87 ± 7.52	973 ± 8.18	1.3 ± 0.54	3.01 ± 3.83
Total	427,788	76.6 ± 12.3	17.7 ± 7.58	965.56 ± 13.31	1.3 ± 0.62	3.16 ± 3.82

### Relative humidity-HFMD relationship curves

**Figure 1:**
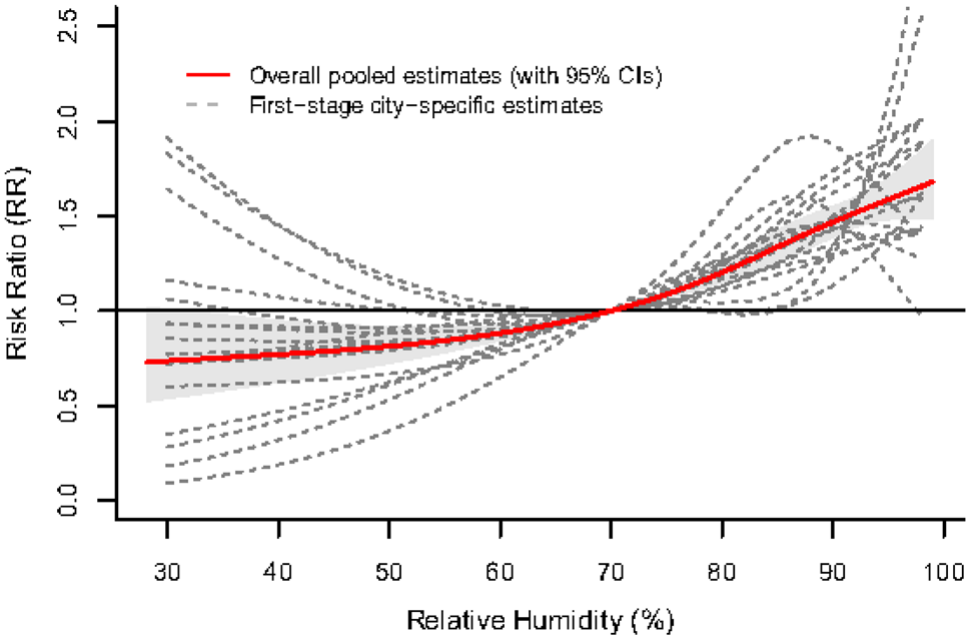
Overall humidity-HFMD relationship curves with 70% relative humidity as the reference value in the Sichuan Basin, 2011–2017.

**Figure 2:**
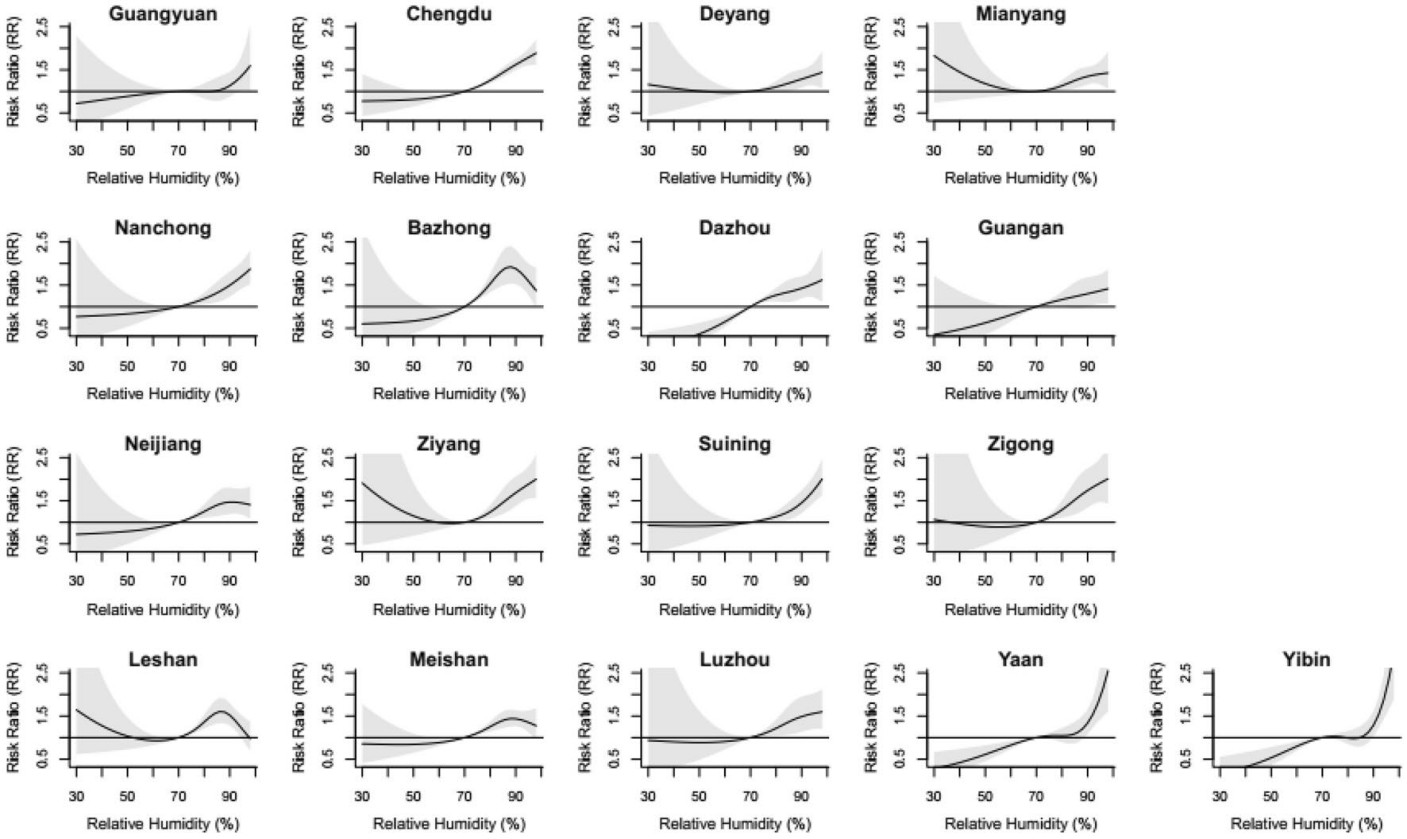
RRs of daily relative humidity on HFMD with 70% as the reference value of relative humidity in the 17 Sichuan Basin cities, 2011–2017. The first to fourth rows are the exposure–response curves for cities in the northwestern, northeastern, central and southern regions, respectively.

Figure 1 shows the overall pooled associations between relative humidity and HFMD for the 17 cities in the Sichuan Basin. The relationship between relative humidity and HFMD presented a J-shaped pattern. The exposure–response curve was initially relatively flat, but once the relative humidity approximately exceeded 70%, the curve began to rise, which indicated that the risk of HFMD increased with the increasing relative humidity. Figure 2 shows the city-specific exposure–response curves. Although we adopted a uniform set of statistical analysis strategies, the effect estimates still varied from city to city. In the northern part of the basin (Bazhong) and in the southern region (Leshan, Meishan), the effect of relative humidity exhibited an approximately inverted V-shaped pattern in the relatively high relative humidity range. The RR increased until it peaked, and then the RR decreased rapidly. In other cities, the curves showed an approximate J-shaped pattern or U-shaped pattern.

### Effect modification results

**Figure 3:**
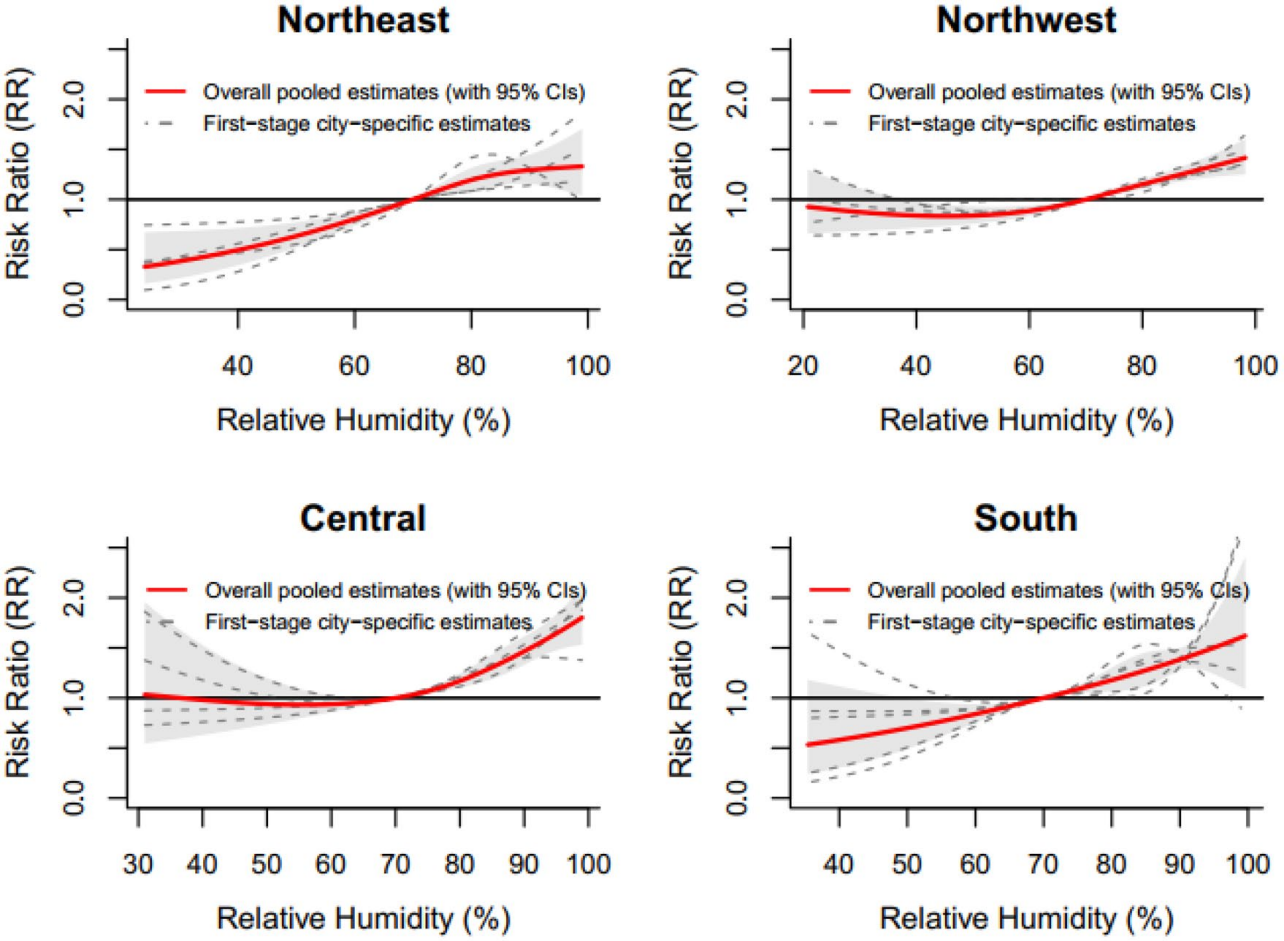
Region-specific pooled humidity-HFMD relationship curves with 70% as the reference value of relative humidity for the 4 regions in the Sichuan Basin, 2011–2017.

**Figure 4:**
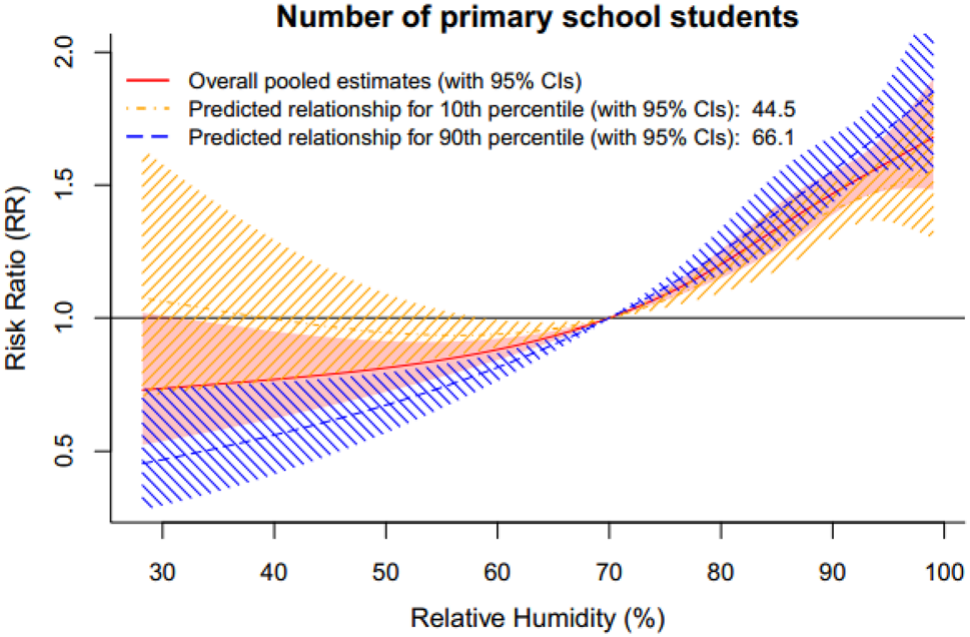
Predicted humidity-HFMD association for the number of primary school students at the 10th and 90th percentiles, with 70% relative humidity as the reference value.

Table 2 summarizes the analysis results of residual heterogeneity. True differences between the 17 cities explained 55.5% of the variation. The results of the multivariate meta-analysis indicated that the heterogeneity was related to the geographical region and the number of primary school students, with the corresponding I^2^ decreasing from 55.5% (intercept-only model) to 52.5% and 51.1%, respectively. We found no significant modification effects for other variables (P value of the LR test > 0.05). Figure 3 shows the relationship curves of the 4 different regions in the Sichuan Basin. Figure 4 indicates that the effects of relative humidity increased as the number of primary school students increased. More results from the stratified analyses are shown in Online Appendix Figures S2–S5.

**Table 2:** Heterogeneity of relative humidity effects and its relationship with city-level variables.

Variables	LR test			Model fit	Cochran Q test			I^2^
	stat	dfs	P	AIC	stat	dfs	P	(%)
Intercept-only model	–	–	–	70.4	107.7	48	< 0.001	55.5
Region	17.82	9	0.037	70.5	82.1	39	< 0.001	52.5
Population density	5.03	3	0.17	71.3	99.5	45	< 0.001	54.8
Population growth	5.24	3	0.155	71.1	97.4	45	< 0.001	53.8
Number of primary school students	10.61	3	0.014	65.7	92	45	< 0.001	51.1
GDP per capita	0.62	3	0.892	75.7	106	45	< 0.001	57.6
GDP growth	6.59	3	0.086	69.8	97	45	< 0.001	53.6
Registered physicians	3.65	3	0.302	72.7	103.2	45	< 0.001	56.4
Hospital beds	4.39	3	0.223	72	103.5	45	< 0.001	56.5
Travel passengers	2.66	3	0.447	73.7	102.7	45	< 0.001	56.2

### Attributable risk results

**Figure 5:**
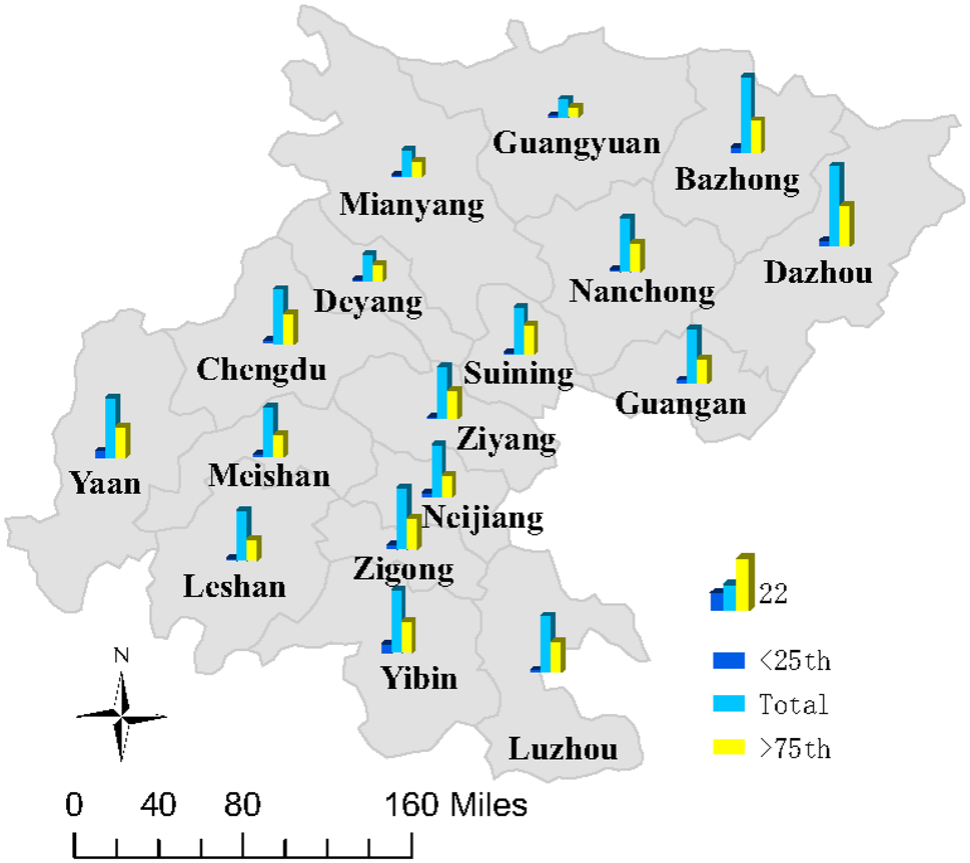
AFs of HFMD for total and individual components for relative humidity in the 17 Sichuan Basin cities. This figure was generated using ArcGIS 10.0 software (https://www.esri.com/software/arcgis/arcgis-for-desktop).

The estimated city-specific ANs and AFs for total relative humidity and high relative humidity above the 75th percentile and low relative humidity below the 25th percentile are shown in Fig. 5. Overall, 118,775 reported HFMD cases were attributed to relative humidity in total. The total AF was 27.77% (95% CI 25.24–30.02%), with Dazhou having the highest AF (43.27%) and Guangyuan having the lowest AF (9.72%) (Online Appendix Table S2). Table 3 shows the total attributable risk and individual components in the 4 regions of the Sichuan Basin. The total AF decreased from 35.72% (95% CI 29.8–40.49%) in the northeastern region to 27.20% (95% CI 23.19–31.23%) in the southern region; however, the northwestern region had the lowest AF, at 25.58% (95% CI 22.08–29.03%). High relative humidity was responsible for the majority of the HFMD burden, with an AN of 62,295 and an AF of 14.56% (95% CI 13.38–15.69%). The fraction attributed to high relative humidity decreased by latitude from 17.31% (95% CI 14.55–19.65%) in the northeastern region to 12.73% (95% CI 10.61–14.58%) in the southern region.

**Table 3:** Attributable risk of HFMD computed as the total and as individual components for relative humidity with 95% eCIs in the 4 regions of the Sichuan Basin.

City	Attributable numbers			Attributable fraction (%, 95% eCI)		
	Total	< 25th	> 75th	Total	< 25th	> 75th
Northeastern region	21,128	1328	10,242	35.72 (29.8, 40.49)	2.24 (1.51, 2.87)	17.31 (14.55, 19.65)
Northwestern region	61,970	3591	34,506	25.58 (22.08, 29.03)	1.48 (1.08, 1.85)	14.24 (12.52, 15.94)
Central region	14,486	821	7628	29.92 (24.02, 35.23)	1.69 (0.86, 2.45)	15.76 (13.01, 18.09)
Southern region	21,191	1564	9962	27.20 (23.19, 31.23)	2.01 (1.34, 2.6)	12.73 (10.61, 14.58)
Overall	118,775	7303	62,295	27.77 (25.24, 30.02)	1.71 (1.42, 1.96)	14.56 (13.38, 15.69)

## Discussion

The results quantified the influence of relative humidity on HFMD in children in the Sichuan Basin from multiple perspectives. This research has two main strengths. To our knowledge, this is the first study conducted in a basin region with diverse climatic and socioeconomic characteristics using a multicity statistical analysis strategy to explore the association between relative humidity and HFMD in children. The complex nonlinear and delayed impacts of relative humidity were firstly included to measure the risk of HFMD in children attributable to relative humidity.

This study evaluated the effects of relative humidity in 17 Sichuan Basin cities through a multicity two-stage time series modeling strategy. Consistent with previous studies in this region, this study found an approximate nonlinear J-shaped relationship, in which the risk of HFMD increased with increasing relative humidity. A study in mainland China^28^ also suggested a monotonic increasing curve in southwestern China. Although research on the exact mechanisms is lacking, some evidence suggests that relative humidity may influence HFMD through several complex pathways. First, enteroviruses, the pathogens of HFMD, can survive in the environment for a period of time, increasing the chance to infect vulnerable individuals^29^. Relative humidity may impact the survival time and reproductive capacity of enteroviruses, thereby increasing or decreasing the chance of contact^30^. Second, on days with high relative humidity, infected people may excrete more enteroviruses into the environment^31^, which could easily attach to the small particles on the surface of toys or in the air^32^. This process could lead to the accumulation of the virus in the environment, thus accelerating the spread of HFMD. Third, humoral or cellular immunity could be affected by climatic factors^33^, especially in children with relatively immature immune systems and poor self-care abilities^34^. As relative humidity increases, body sweats and metabolism are restricted, making children more susceptible to the enteroviruses that cause HFMD. Therefore, the impact of relative humidity should be taken into account for future control and prevention policies for HFMD.

In this study, a distributed lag nonlinear model (DLNM) combined with a multivariate meta-regression model was applied to conduct a unified analysis of multicity data to eliminate the possible impact of heterogeneity due to methodology. The I^2^ statistics from the Cochran test show moderate heterogeneity among the variables, which is consistent with most previous studies^12,23^. Among all city-level variables, geographical region and the number of primary school students remained the two main potential effect modifiers leading to significant heterogeneity. Increased susceptibility to HFMD has been identified in the southern basin, which has high relative humidity. A previous study conducted in Sichuan Province suggested that the dominant HFMD serotypes also showed obvious regional differences, which varied from 2011 to 2017^35^. Different strains of pathogens may have different behaviors due to relative humidity^30^. In the southern basin, under the condition of high relative humidity, pathogens may be able to thrive depending on humidity, resulting in longer survival times, and have stronger infectiousness. Further research is required to investigate regional differences in pathogen-specific performance depending on meteorological factors in the time scale. Moreover, previous virological studies reported that temperature plays an important role in the survival of enteroviruses and can affect the daily activities of the host and the excretion of enteroviruses^31,36^. The southern basin tends to experience relatively high temperatures, increasing the effect of high relative humidity on HFMD^37,38^, which may be another mechanism of the impact of relative humidity on HFMD. Similar to the findings of the present research, a study of 8 cities in Guangdong Province^23^ found that geographic location indicators, such as longitude and latitude, partially explained the heterogeneity of results among cities. Similarly, the effects of regional differences in humidity on HFMD were also observed in the second peak, especially in northwest and south regions, which show increased susceptibility to HFMD. More comprehensive stratified analyses are still needed to examine the season-stratified effects of meteorological factors such as relative humidity on childhood HFMD.

On the other hand, primary school students, the crucial HFMD-susceptible group, gather in fixed public places, such as schools, for a long time. More students results in closer the contact between susceptible students and infected students, consequently promoting the spread of HFMD. Notably, GDP growth was also fairly close to reaching significance. On one hand, people who are more economically well off may have greater health awareness and a limited need for extra day care, with higher socioeconomic status therefore corresponding to lower susceptibility^39^. On the other hand, children in developed areas with better infrastructure and public transport may also tend to engage in more outdoor activities and have more social contact, thus causing higher HFMD burdens^40^.

Our results revealed that humidity above the 75th percentile of relative humidity accounted for more than 50% of humidity-related HFMD cases, which is far higher than the proportion of 6% with humidity below the 25th percentile of relative humidity, indicating that the relative humidity-related attributable risk of HFMD primarily depends on relatively high humidity. Therefore, policymakers should increase parents’ awareness of the adverse health effects of wet environments and allocate additional health resources to implement effective public health interventions, especially for children in the northern basin. In addition, the AF attributable to relative humidity varied from 9.72 to 43.27% among cities. In general, the AF associated with high relative humidity in the northern basin was higher than that in the southern basin, while the AF of low relative humidity was higher in the southern basin. Compared with children in the southern basin, children in the northern basin are more susceptible to HFMD under high relative humidity conditions. One explanation is that people change their physical appearance and individual behaviors to adapt to local environmental conditions. The popularity of humidity control devices can help partly reduce the impact of high and low humidity on HFMD infections in children.

This study has several limitations. First, this was an ecological study, and the meteorological data were obtained from fixed monitoring stations rather than the real exposure of individuals. Therefore, measurement error is inevitable and may be randomly distributed, consequently underestimating the humidity-HFMD association^41^. Second, this study took city-level geographic, demographic, economic, health resource and traffic variables into account, but they accounted for only a small part of the heterogeneity. Some other important meta-predictors may also be related to HFMD, such as vegetation coverage and prevention and control measures for infectious diseases, which may partly explain the heterogeneity. However, due to limited access to data, these factors were not considered in this study. In addition, modeling the relationship between weather and infectious diseases while controlling residual autocorrelations by including an autoregressive term of past case counts into time-series regression models remains controversial. Some studies believe that lagged case counts are needed to control autocorrelations^42^, while other studies argue that including lagged case counts into the causal path of the overall weather-lag-disease relationship would result in underestimation of the true relationship^43,44^. In this study, we did not include the autoregressive term in the base model to avoid a possible downward bias.

In conclusion, this study reveals a J-shaped relationship between relative humidity and HFMD in the Sichuan Basin, though the relationship varied from city to city. Geographic region and number of primary school students were the two primary effect modifiers. High relative humidity contributed to most of the HFMD burden attributable to relative humidity. Our findings can help policy makers and parents improve the negative health effects of low and high relative humidity on child morbidity due to HFMD. These research findings may also provide an important basis from multiple perspectives for relevant public health decision-making and allow the allocation of additional health resources to mitigate the adverse health impacts of relative humidity, especially for people living in regions with relatively high humidity.

## Methods

### Study site

The Sichuan Basin, located in southeastern China, covers a total of 185,757 km^2^. Seventeen cities with a population of over 82 million are present in Sichuan Basin (according to 2014 demographic data). According to the national standard of meteorological and geographic division, the Sichuan Basin is divided into 4 regions (see Online Appendix Figure S1)^45^. The Sichuan Basin has a subtropical monsoon climate, with annual precipitation ranging from 1000 to 1300 mm. The precipitation is not uniform among regions in the Sichuan Basin; the greatest amount of precipitation occurs in the mountainous lands at the edge of the Sichuan Basin. Precipitation is also unevenly distributed throughout one year, with 70–75% of the rainfall concentrated in summer (June–October).

### Data collection

HFMD was listed as a class “C” notifiable disease for reporting and management in China in 2004. Each HFMD case clinically diagnosed according to a standard guideline should be reported online within 24 h^46^. City-specific clinical HFMD case information, including basic demographic information (e.g., sex and date of birth), disease information (e.g., date of symptom onset) and report information (e.g., date of entry), was obtained from the China Information System for Disease Control and Prevention in the Sichuan Basin from 1 January 2011 to 31 December 2017. The China Center for Disease Control and Prevention assured and assessed the quality of the dataset. Among the dataset, children aged 0–15 years accounted for more than 99.7% of HFMD cases. Therefore, we selected reported HFMD cases in children under 15 years old throughout the Sichuan Basin for further analysis. Finally, we aggregated all cases under the age of 15 with symptom presentation into the daily HFMD counts at the prefecture level in 17 cities in the Sichuan Basin.

Daily meteorological surveillance data, including the mean relative humidity (%), mean temperature (℃), minimum temperature (℃), maximum temperature (℃), mean atmosphere pressure (hPa), mean wind velocity (m/s) and sunshine duration (h), were collected from the China Meteorological Data Sharing Service System. For humidity, temperature, atmosphere pressure and wind velocity, the missing values were imputed using linear interpolation; for sunshine duration, the missing values were filled by zero depending on the nature of meteorological data. However, the missing data issue should be negligible given the very small proportion of missing values (approximately 0.1%) and the high quality of the monitoring data. For cities with no meteorological monitoring stations within the city administrative boundaries, data from the station in adjacent cities closest to the city center were used^47^. For cities with more than one meteorological monitoring station, we selected data from the station closest to the city geometric center^12,14^.

We obtained city-level geographic characteristics (region), demographic characteristics (population density, population growth and number of primary school students), economic characteristics (gross domestic product (GDP) per capita and GDP growth), health resource characteristics (number of registered physicians and number of hospital beds) and traffic characteristics (travel passengers) from the China City Statistical Yearbooks from 2011 to 2017. For each city, the arithmetic averages of all the city-level variables were calculated as a proxy for socioeconomic differences among cities in the Sichuan Basin. In the second-stage analysis, these average variables were added as modifiers to explore modification effects on the humidity-HFMD relationship.

### Statistical analysis

We used a two-stage multicity time series analysis strategy to analyze data from the Sichuan Basin from 2011 to 2017. First, a distributed lag nonlinear model (DLNM) was applied for each city’s data to obtain city-specific effect estimates. Second, we applied a multivariate meta-analysis to combine the effect estimates obtained from the first-stage analysis, and city-level variables were included to further investigate whether they could explain the heterogeneity between cities. Then, the risk of HFMD attributed to relative humidity was calculated by a backward perspective method.

#### City-specified distributed lag nonlinear model

For each city, we constructed a DLNM with a quasi-Poisson distribution to measure the nonlinear and lag relationship between relative humidity and HFMD^48^. A cross-basis function was used to describe the two-dimensional exposure-lag-response association. We used natural cubic splines for the effects of relative humidity with 3 degrees of freedom (dfs) and natural cubic splines for lag effects with 4 dfs. Based on the parametrization in previous studies^12,15,37^ and the incubation period (approximately 3–5 days)^46^, we chose 3–17 days as the lag range to study the lag structure of the humidity-HFMD relationship. Mean temperature, atmospheric pressure, sunshine duration, and wind velocity were important confounding factors in the humidity-HFMD relationship. Therefore, we controlled for temperature by calculating simple moving weighted averages in the same lag range as relative humidity and a natural cubic spline with 3 dfs. The other three factors were controlled by calculating exponential moving weighted averages in the same lag range as relative humidity. A natural cubic spline with eight dfs per year was used to control for seasonality and long-term trends of HFMD. The day of week and holidays were set as two indicator variables in the model.

#### Second-stage analysis

For the second-stage analysis, to obtain the overall effect estimates of relative humidity in the Sichuan Basin, we reduced the estimates from DLNMs and then fitted an intercept-only multivariate meta-regression model^49,50^. Based on the intercept-only model, we individually included city-level variables in the intercept-only model to construct univariable meta-regression models to explore effect modifications. The likelihood ratio (LR) test was applied to test the significance of these variables. We identified and quantified residual heterogeneity through multivariate extension of the Cochran Q test and I^2^ statistic^50^. Stratified analyses were conducted for the first peak (April–July) and the second peak (September–December) to further examine whether regional differences in humidity effects on HFMD existed in two peaks periods.

#### Calculation of attributable risk

The minimum-morbidity relative humidity, corresponding to the relative humidity associated with the minimum morbidity risk between the 5th and 95th percentiles^51^, was considered the counterfactual exposure reference to calculate the infection burden of HFMD attributable to relative humidity. City-specific minimum-morbidity relative humidity was identified from the city-specific exposure–response relationship curve. We adopted a backward perspective considering that current risk was the accumulation of the exposure effect of a previous period^52^. We summed the contributions from all days in the series to compute the total number of HFMD cases attributed to relative humidity. The ratio of the number to the actual total cases in the corresponding city or region is the AF. We calculated the components attributable to high relative humidity above the 75th percentile and low relative humidity below the 25th percentile. Empirical confidence intervals (eCIs) were derived by Monte Carlo simulations^52^.

Considering the systematic sensitivity analysis strategy proposed by Xiao^12^ and prior knowledge (see more details in Online Appendix Text S1), we determined the model parameters and evaluated the robustness of the model. A two-tailed P value of < 0.05 was considered statistically significant. All models were fitted with R3.5.1 using the “dlnm” package to construct the DLNMs and the “mvmeta” package to perform the multivariate meta-analysis. All geographic maps were constructed using ArcGIS 10.0.

### Ethics statement

All HFMD surveillance data were collected from the China Information System for Disease Control and Prevention. This study was analyzed at the aggregate level and the research protocol was approved by the institutional review board of the School of Public Health, Sichuan University. All methods were carried out in accordance with relevant guidelines and regulations. All of the patients’ records were anonymized and deidentified, thus the need for informed consent was waived by the Ethics Review Committee of the Sichuan Provincial Center for Disease Control and Prevention.

## Supplementary information

Supplementary Information.

## Data Availability

The datasets used and/or analyzed during the current study are available from the corresponding author on reasonable request.
