# Supplementary material for: The burden of childhood hand-foot-mouth disease morbidity attributable to relative humidity: a multicity study in the Sichuan Basin, China
Source: Sci Rep. 2020 Nov 10;10:19394. doi: 10.1038/s41598-020-76421-7 (PMC7656260; doi:10.1038/s41598-020-76421-7)
Supplement: Supplementary file 1 — Supplementary Information. [file 41598_2020_76421_MOESM1_ESM.pdf]

The burden of childhood hand-foot-mouth disease morbidity attributable to relative humidity: A multicity study in the Sichuan Basin, China

Caiying Luo [1]†, Yue Ma[1]†, Yaqiong Liu[2], Qiang Lv[2] & Fei Yin[1]*

[1] West China School of Public Health and West China Fourth Hospital, Sichuan University, Chengdu, Sichuan, China. [2] Sichuan Center for Disease Control and Prevention, Chengdu, Sichuan, China.

†: These authors contributed equally to this work and should be considered co-first authors.

*Corresponding author

Figures

Figure S1. Geographical locations, relative humidity and the numbers of HFMD cases in 17 cities in the Sichuan Basin of China. The maps were generated using ArcGIS 10.0 software (http://www.esri.com/software/arcgis/arcgis-for-desktop).

Figure S2. Time series plot for daily cases of HFMD in the Sichuan Basin from 2011 to 2017.

Figure S3. The stratified analyses of humidity effects by peaks.

Figure S4. The stratified analyses for regional differences in humidity effects in the first peak.

Figure S5. The stratified analyses for regional differences in humidity effects in the second peak.

Tables

Table S1 Description of socioeconomic characteristics in the Sichuan Basin from 2011 to 2017.

Table S2 Attributable risk of HFMD infection computed as total and as individual components for relative humidity with 95% eCIs in the 17 Sichuan Basin cities.

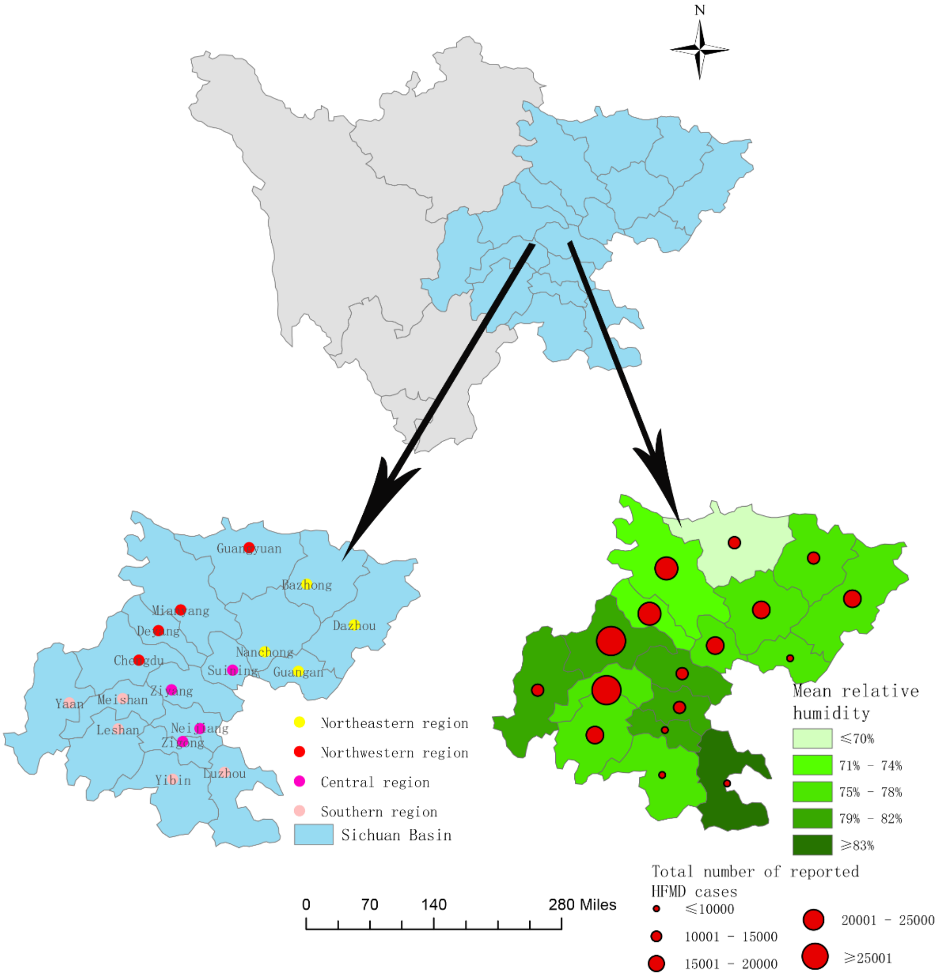

Figure S1. Geographical locations, relative humidity and the numbers of HFMD cases in 17 cities in the Sichuan Basin of China. The maps were generated using ArcGIS 10.0 software (http://www.esri.com/software/arcgis/arcgis-for-desktop).

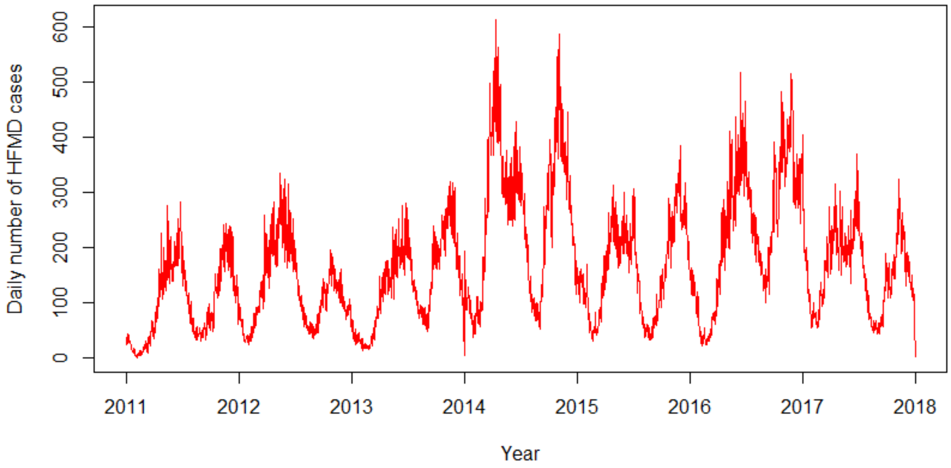

Figure S2. Time series plot for daily cases of HFMD in the Sichuan Basin from 2011 to 2017.

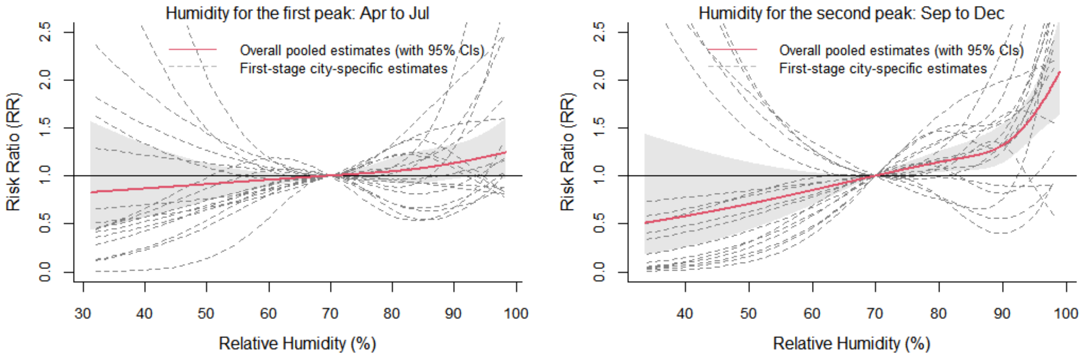

Figure S3. The stratified analyses of humidity effects by peaks.

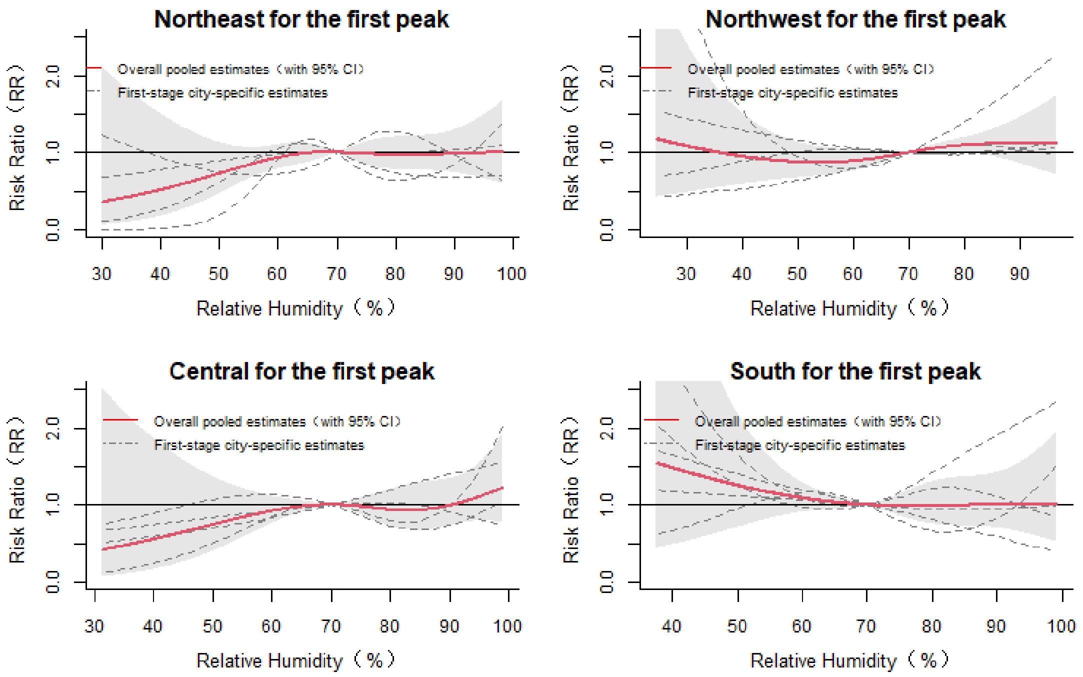

Figure S4. The stratified analyses for regional differences in humidity effects in the first peak.

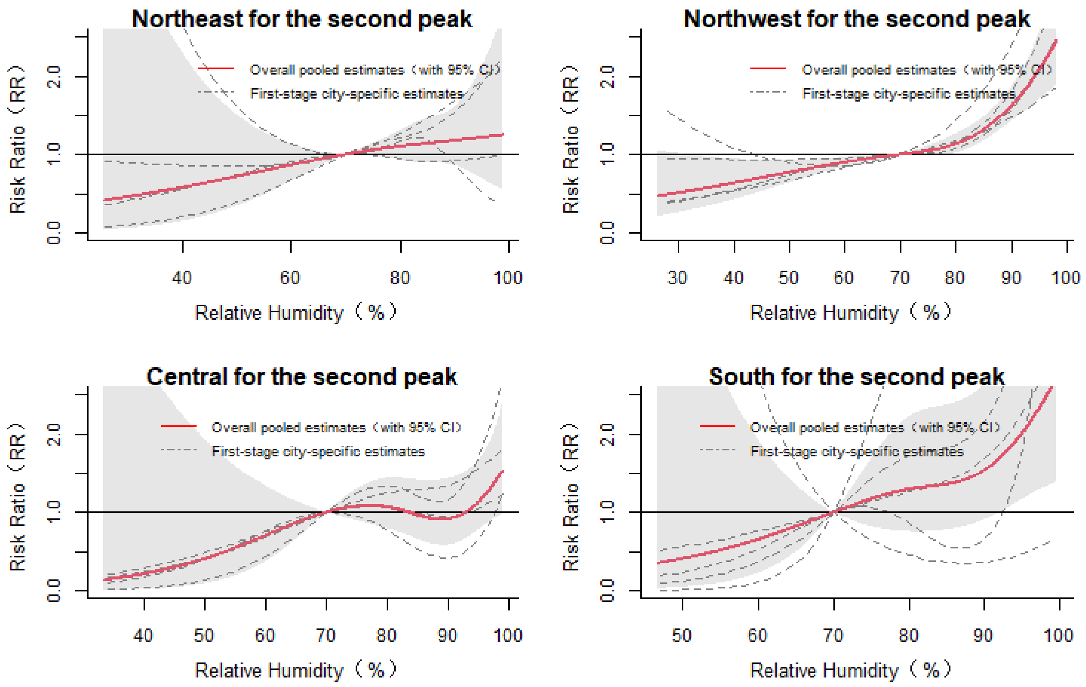

Figure S5. The stratified analyses for regional differences in humidity effects in the second peak.

Table S1 Socioeconomic characteristics in the Sichuan Basin from 2011 to 2017.

City	Region	Population density (/km^2)	Population growth (%)	Number of primary school students (per 1000)	GDP per capita (yuan)	GDP growth (%)	Number of registered physicians (per 1000)	Number of hospital beds (per 1000)	Travel passengers (per 10000)
Chengdu	Northwestern	985	0.331	62.6	72580	10.474	3.9	8.1	56786.6
Zigong	Central	748	0.307	53.3	39136	11.284	1.1	4.6	7234.3
Luzhou	Southern	414	0.474	78.9	31663	11.797	1.7	4	10143.4
Deyang	Northwestern	661	0.067	42.8	44376	10.263	1.9	4.4	8545.7
Mianyang	Northwestern	269	0.284	45.8	35691	10.303	2	5.1	7808
Guangyuan	Northwestern	189	0.054	49	21988	10.667	1.7	5	6659.1
Suining	Central	710	0.024	45.6	25386	11.106	1.9	4	4555.1
Neijiang	Central	785	0.223	54.3	29837	10.044	1	4.3	12742.4
Leshan	Southern	278	0.123	48.6	38503	10.377	2	4.6	6592.6
Nanchong	Northeastern	601	0.104	53.2	935257	10.417	1.3	3.7	10172
Meishan	Southern	490	-0.003	41.9	32077	10.727	1.4	3.7	6220.6
Yibin	Southern	415	0.37	67.5	34515	10.23	1.3	4.1	10524.3
Guangan	Northeastern	739	0.457	54.7	27661	11.017	1	2.6	48062.7
Dazhou	Northeastern	414	0.469	65.2	22936	9.34	1	3.1	8780.6
Yaan	Southern	104	0.181	59.7	30238	10.143	2.3	6.4	2539.1
Bazhong	Northeastern	312	0.349	59	13780	9.934	1.9	3.4	5401
Ziyang	Central	629	0.05	53.4	33094	10.89	1	3.6	6175
Total		514	0.227	55	86395	10.53	1.7	4.4	12879

Table S2 Attributable risk of HFMD infection computed as total and as individual components for relative humidity with 95% eCIs in the 17 Sichuan Basin cities.

City	ANs			AFs (%, 95% empirical CI)		
	Total	<25th	>75th	Total	<25th	>75th
Chengdu	54163	3206	29889	29.33 (25.3,33.37)	1.74 (1.29,2.16)	16.18 (13.99,18.29)
Zigong	2609	182	1310	33.08 (18.88,45.04)	2.31 (-0.25,4.62)	16.61 (10.79,21.7)
Luzhou	2544	95	1387	30.19 (10.53,44.52)	1.13 (-0.56,2.66)	16.46 (7.51,24.45)
Deyang	3212	100	1992	14.23 (2.79,23.4)	0.44 (-0.49,1.33)	8.83 (3.56,13.49)
Mianyang	3580	178	2068	14.55 (6.1,21.95)	0.72 (-0.22,1.71)	8.4 (4.75,11.94)
Guangyuan	1015	107	557	9.72 (-10.28,26.7)	1.02 (-2.33,4.06)	5.34 (-1.11,11.05)
Suining	4006	147	2511	24.88 (12.72,34.59)	0.91 (-0.59,2.33)	15.59 (10.16,20.1)
Neijiang	4010	346	1702	28.25 (17.23,36.6)	2.44 (0.8,4.02)	11.99 (7.07,16.17)
Leshan	4277	167	1845	26.76 (17.91,34.66)	1.04 (0.61,1.41)	11.55 (7.25,15.63)
Nanchong	5761	219	3053	29.07 (17.53,38.5)	1.11 (-0.26,2.32)	15.41 (10.75,19.15)
Meishan	8079	485	3501	26.33 (18.59,32.56)	1.58 (0.5,2.7)	11.41 (8.18,14.4)
Yibin	3040	445	1527	33.79 (20.35,44.75)	4.95 (2.15,7.68)	16.98 (11.68,21.76)
Guangan	2803	175	1252	29.42 (14.76,41.67)	1.83 (0.24,3.35)	13.14 (6.9,19.21)
Dazhou	7062	520	3591	43.27 (33.1,51.75)	3.19 (1.89,4.35)	22 (15.57,27.28)
Yaan	3253	372	1659	31.81 (19.2,42.27)	3.63 (1.65,5.51)	16.23 (10.74,20.69)
Bazhong	5502	414	2347	40.79 (31.19,48.23)	3.07 (1.82,4.17)	17.4 (13.21,21.27)
Ziyang	3860	146	2105	27.9 (17.65,36.13)	1.05 (0.3,1.7)	15.22 (10.74,19.02)
Overall	118775	7303	62295	27.77 (25.24,30.02)	1.71 (1.42,1.96)	14.56 (13.38,15.69)

Text S1. Sensitivity Analysis

1. The choice of degrees of freedom to control long-term trends and seasonality.

To control long-term trends and seasonality, we performed a sensitivity analysis to change the dfs from 1 to 15 per year. The results are presented in Figure S6. When the dfs of natural cubic splines exceeded 8, both the average estimates of the relative humidity effect and goodness of fit remained stable. Therefore, we set the dfs per year to 8 to control long-term trends and seasonality.

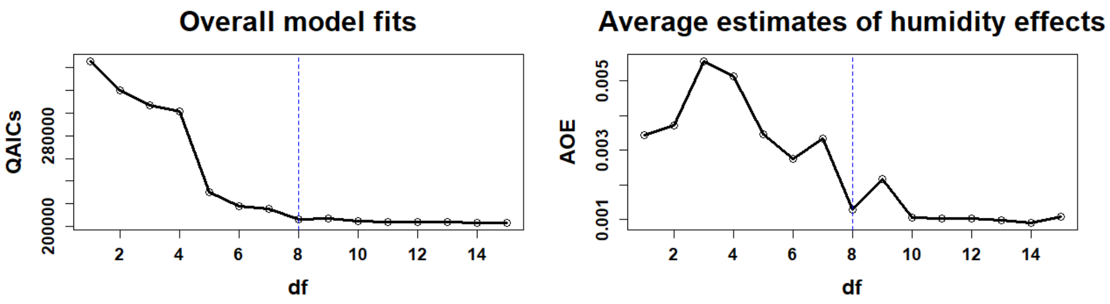

Figure S6. The overall model fits (left panel) and average estimates of humidity effects (right panel) for different dfs of time splines.

2. The choice of the reference value of relative humidity.

We performed an alternative analysis with references values of 75%, 80%, and 40%. As shown in Figure S7 and Table S3, all the shapes of the humidity-HFMD curves and the main results of modification effects in the second-stage analysis were robust. We used 70% humidity as the reference value mainly based on the following reasons. Firstly, most studies have used the mean or median value of exposure as a reference to achieve higher statistical power and narrower confidence intervals. In our study, the range of the mean relative humidity was 70%-80% in the 17 cities (Main text: Table 1). According to the results of the sensitivity analysis, setting references in this range yielded the same shapes of exposure-response curves and narrower confidence intervals than other references such as 40%. Secondly, the exposure-response curve was initially relatively flat. However, once the relative humidity exceeded approximately 70%, the curve began to rise, indicating that the risk of HFMD increased with increasing relative humidity (Main text: Figure 1). Therefore, we used 70% humidity as the reference value.

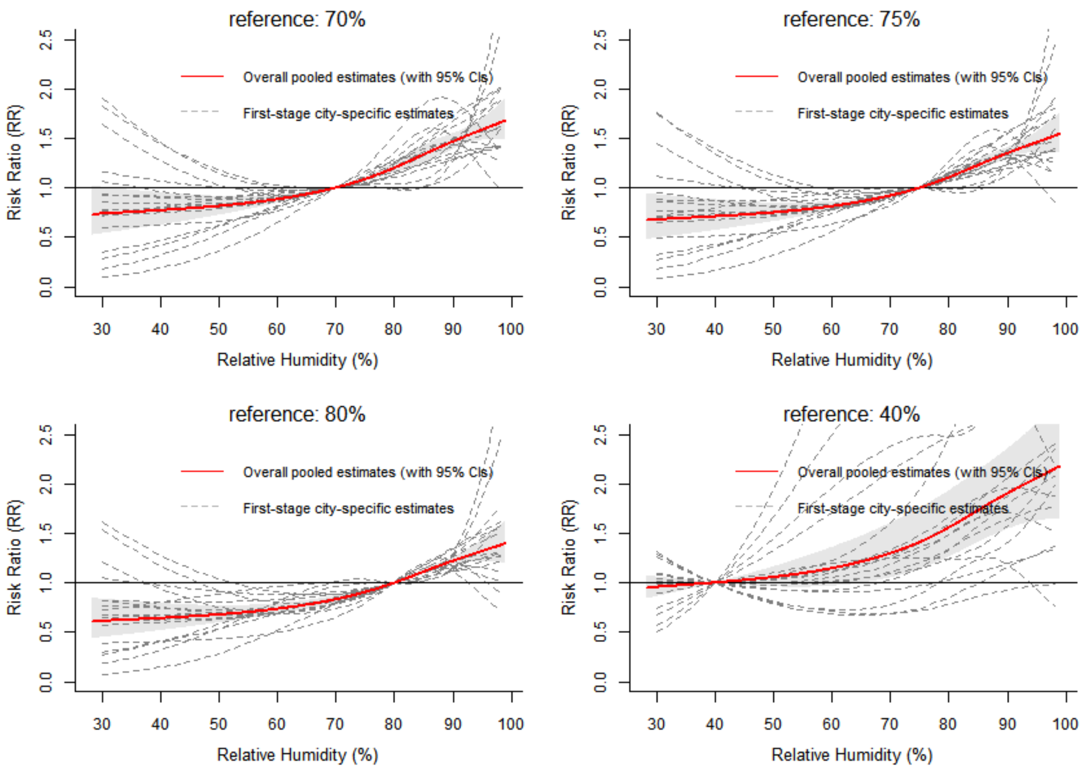

Figure S7. Overall humidity-HFMD relationship curves with 70%, 75%, 80%, and 40% relative humidity as the reference values.

Table S3. Modification of city-level variables on relative humidity effects and model fits.

Variables	LR test			Model fit
	stat	dfs	P	AIC
Intercept-only model	—	—	—	70.4
Region	17.82	9	0.037	70.5
Population density	5.03	3	0.17	71.3
Population growth	5.24	3	0.155	71.1
Number of primary school students	10.61	3	0.014	65.7
GDP per capita	0.62	3	0.892	75.7
GDP growth	6.59	3	0.086	69.8
Registered physicians	3.65	3	0.302	72.7
Hospital beds	4.39	3	0.223	72
Travel passengers	2.66	3	0.447	73.7

* the results of the multivariate meta-regression analysis (reference values of 75%, 80% and 40%) remained the same because varying only the reference value did not influence the estimations.

3. Controlling for measured time-varying confounders.

For each confounder, we compared 9 different models by varying the association pattern (i.e., linear or nonlinear) and lag structure (i.e., how many lags should be included). Table S4 shows the detailed settings of the confounder models. C0 is the null model with no confounder. C1-C4 are based on the assumption of a linear relationship of the association pattern. C1 includes only one lag, which is set to the median of the incubation period. C2 to C4 include multiple lags but use different methods to summarize the multiple lags through simple and exponential moving weighted averages and distributed lag models. A natural cubic spline with 4 dfs is applied to constrain the distributed lag model. Similarly, C5-C8 are based on the assumption of a nonlinear relationship of the association pattern. A natural cubic spline with 3 dfs is applied to describe the nonlinear relationship. The average estimates of humidity effects and model fits are presented in Figures S8-S11. As shown in Figure S8, for the mean temperature results, the confounder model (C6) based on the assumption of a nonlinear relationship with multiple lags and simple moving weighted averages has the best model fit. Similarly, for the other three confounders, we simultaneously considered the parsimony and the consistency of controlling for the time-varying confounders. Finally, we controlled these factors (atmospheric pressure, sunshine duration, and wind velocity) by calculating exponential moving weighted averages in the same lag range as relative humidity.

Table S4. Different confounder model settings.

Notation	Confounder model	Notation	Confounder model
C0	No confounder		-
C1	Linear relationship (lag 4)	C5	Nonlinear relationship (lag 4)
C2	Linear relationship (lags 4-10)<br>Simple moving average	C6	Nonlinear relationship (lags 4-10)<br>Simple moving average
C3	Linear relationship (lags 4-10)<br>Exponential moving average	C7	Nonlinear relationship (lags 4-10)<br>Exponential moving average

C4	Linear relationship (lags 4-10)<br><br>Distributed lag model	C8	Nonlinear relationship (lags 4-10)<br><br>Distributed lag model

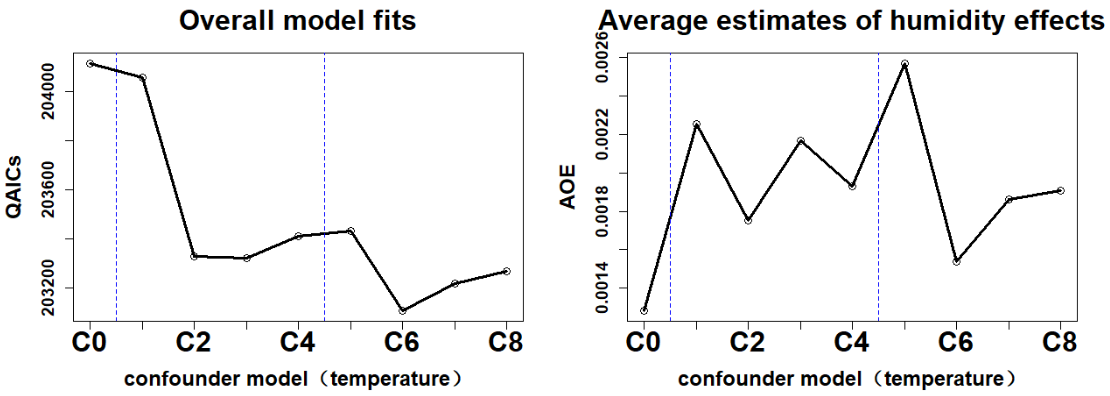

Figure S8. Overall model fits (left panel) and average estimates of humidity effects (right panel) in the 17 cities for different confounder model settings (the mean temperature).

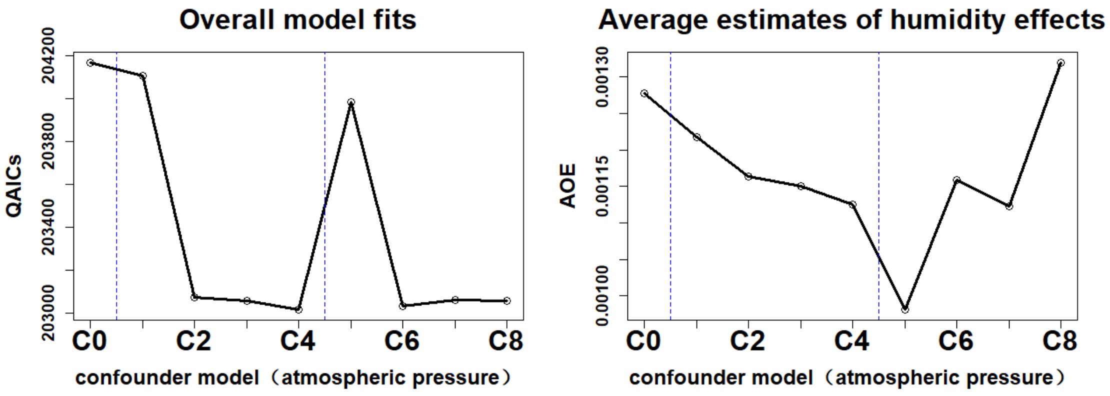

Figure S9. Overall model fits (left panel) and average estimates of humidity effects (right panel) in the 17 cities for different confounder model settings (atmospheric pressure).

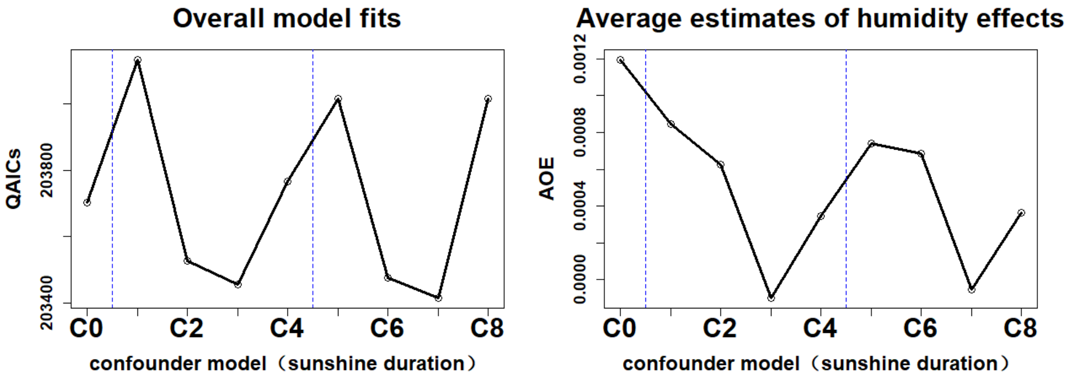

7

Figure S10. Overall model fits (left panel) and average estimates of humidity effects (right panel) in the 17 cities for different confounder model settings (sunshine duration).

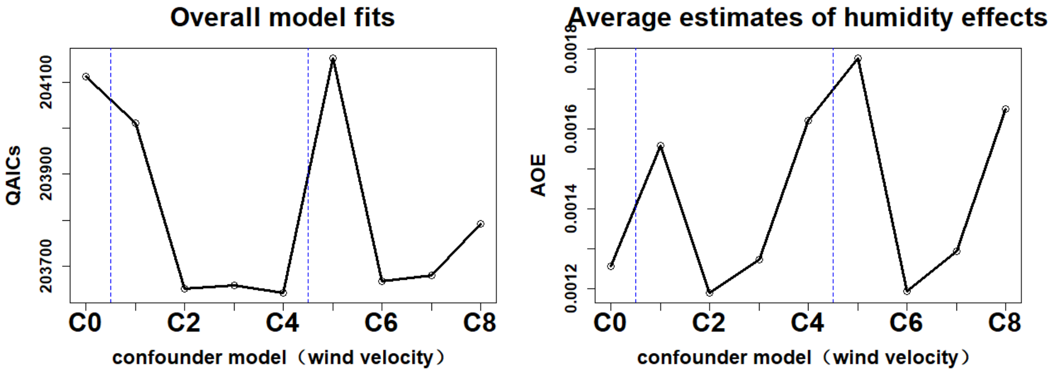

Figure S11. Overall model fits (left panel) and average estimates of humidity effects (right panel) in the 17 cities for different confounder model settings (wind velocity).

4. The choice of the lag structure of relative humidity effects on HFMD.

To explore the whole lag structure of humidity effects, we used a series of lag settings up to 30 days to construct an unconstrained distributed lag model for the 17 cities. According to the results shown in Figure S12, we chose a max lag of 17 days for two main reasons. Firstly, as shown in Figure S12, the average estimates of humidity effects after the lag of 17 days for the 17 cities gradually decreased to zero and then regularly fluctuated around zero, which may be negligible. Secondly, considering the incubation period (3-5 days) and the approximately two-week infection period for HFMD, 17 days was chosen to reflect the lag structure of humidity effects on HFMD based on the natural history of HFMD infection. Splines with 3-5 dfs were commonly used to capture the lag distribution when modeling the relationship between weather and infectious diseases. As our main focus is shaping the exposure-response relationship instead of the lag-response relationship, we chose natural cubic 4-dfs splines, which seem to be sufficient to express the complexity of the lag distribution according to the lag structure figure (Figure S12).

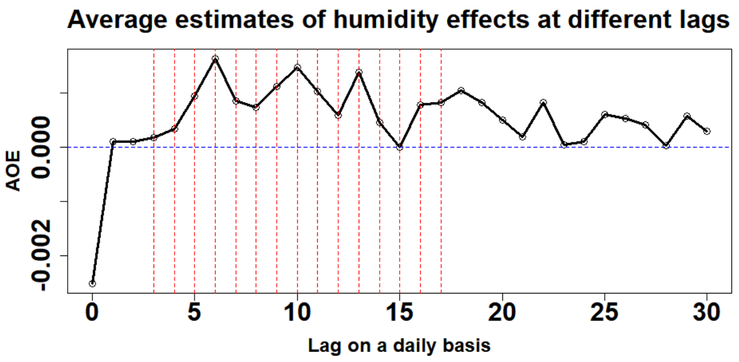

Figure S12. Average estimates of relative humidity effects at different lags (up to 30 days).

5. The choice of the dfs of splines that define the relative humidity-HFMD relationship.

We performed a systematic sensitivity analysis to determine the dfs of natural cubic splines for the effects of relative humidity by varying the dfs from 3 to 8. The results are presented in Figure S13. Natural cubic splines for the effects of relative humidity with 3 dfs show the best goodness of fit, and different dfs yielded robust shapes of the humidity-HFMD curve. Therefore, we used natural cubic splines for the effects of relative humidity with 3 dfs in the final model.

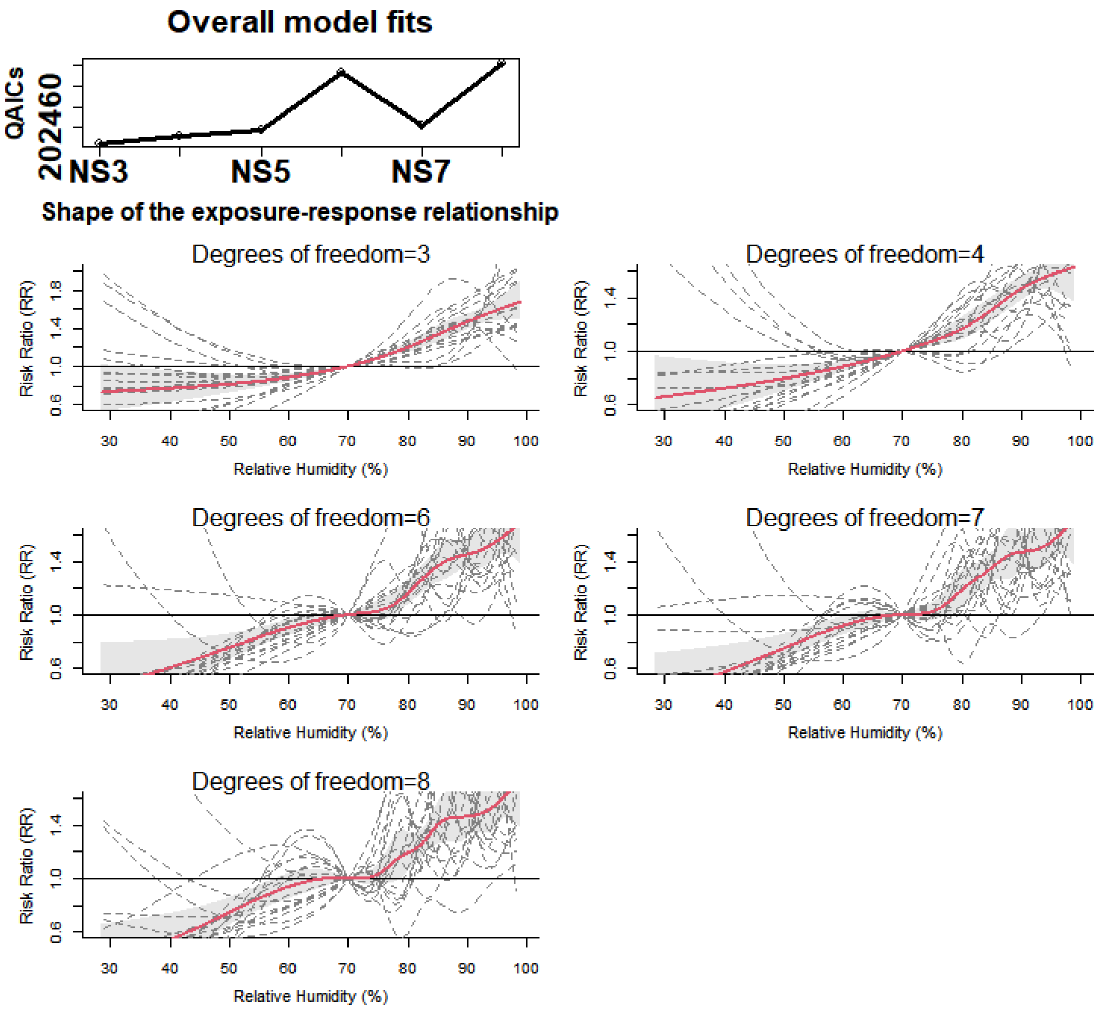

Figure S13. Overall model fits and humidity-HFMD relationship curves for different dfs settings of the exposure-response association.
